# Pediatric radiography entrance doses for some routine procedures in three hospitals within eastern Nigeria

**DOI:** 10.4103/0971-6203.39422

**Published:** 2008

**Authors:** N. O. Egbe, S. O. Inyang, O. B. Ibeagwa, N. O. Chiaghanam

**Affiliations:** Department of Radiography, University of Calabar, Nigeria; 1 Department of Physics, University of Calabar, Nigeria

**Keywords:** Entrance surface dose, exposure, kilovoltage, pediatric, radiography

## Abstract

A survey of the entrance surface doses in the routine radiography of children in eastern Nigeria has been carried out in three hospitals, using thermoluminescence detectors. Chest, abdomen, lumbar spine, skull and pelvis were covered in this study. Findings reveal that doses are higher than the recommended reference values elsewhere, as well as values reported for Sudan. The mean percentage difference in entrance doses for chest radiography for this study and an earlier one carried out for three hospitals in the west of Nigeria is about 44.7%. The high doses are traceable to a lack of standardization in procedure, resulting in use of low tube voltages and high currents for examination, as well as the status of facilities in the area. Recommendations are made for immediate corrective measures to lower the doses.

## Introduction

Clinical procedures in radiography utilize radiation exposures given at different rates. Effects of ionizing radiation on human tissue necessitate the development of strategies for radiation protection in all fields of human endeavor. The International Commission for Radiological Protection (ICRP) recommends that medical activities involving ionizing radiation should fulfill the two basic principles of justification and optimization.[1,2] One of the requirements of the optimization process is regular periodic monitoring of the performance of radiological equipment and assessment of techniques employed in their use. The focus of such monitoring serves to maintain standards, once developed, in equipment performance, image quality and, very importantly, patient doses. It seeks to establish values of measured quantities above which corrective action needs to be taken.[3] Radiation protection of the patient is so highly priced that several regulatory bodies have carried out studies leading to establishment of standards and regulations to guide its practice.[1,3–6] In Nigeria, local legislation has recently given impetus to the regularization of this practice.[7]

Entrance surface exposures (ESE) have been used to report patient doses, and this has been studied for both adult and pediatric patients in many parts of the world.[8–13] Nigeria has not recorded similar evidence of research in patient radiation concerns, especially with regards to children, who have greater susceptibility to radiation effects.[14,15] Entrance skin doses (ESDs) have been reported for some radiological procedures for the adult Nigerian patient.[16–18] These studies are however not widespread, perhaps due to high cost involved in covering the entire country, the lack of adequate monitoring facilities and the generally low level of interest among users of ionizing radiation.

There is paucity of information on pediatric ESDs in the country. To the best of our knowledge, the only existing evidence of any study in this regard[19] is as limited in spread as the preceding adult studies, probably for the reasons earlier stated. This study faced the same logistic problems, for which reason it was limited to the southeastern block of Nigeria, covering three tertiary hospitals with X-ray facilities for general-purpose radiography. All three are major centers of medical activity, but none of them has dedicated facilities for pediatric radiology examinations. The hospitals - the University of Calabar Teaching Hospital (UCTH), Nnamdi Azikiwe University Teaching Hospital (NAUTH) and the Federal Medical Centre (FMC), Owerri - are all located in state capitals within the southeastern block of Nigeria.

The study is the first attempt at measuring ESDs in children in the area of study and will, with the information obtained earlier,[19] add to the pool of data available in national records for general use. It will provide guidance on where efforts on dose reduction will need to be directed to fulfill the requirements of the optimization process and serve as a reference for future work, as well as provide information for comparison with patients of the same category in other countries.

## Materials and Methods

One hundred ninety-five children between the ages of 1 day and 12 years who presented in the hospitals for radiological examination were monitored in the study, using Lithium Fluoride thermoluminescent dosemeter (LiF TLD -100) phosphors for dose measurement. Summary of the radiographic equipment, patient distribution, as well as the average radiographic parameters recorded in each hospital for respective examinations, are as given in Tables 1 and 2 respectively. There was no information or record of quality checks on any of the facilities used in this study. The output of each unit could not be determined as there was no ionization chamber available. The survey was carried out despite this because the aim was to obtain data of entrance surface doses in the area. The commonest routine examinations which exclude the use of contrast media were investigated in the study. The number of patients monitored for each region examined came to 99, 15, 17, 34 and 30 for chest (AP/PA), abdomen (AP), lumbar spine (AP), skull (AP/lateral) and pelvis (AP) respectively. Three TLD chips were attached to the skin of each patient on the path of the primary X-ray beam for all examinations.

**Table 1:** X-ray equipment parameters in the centers studied

*Parameter*	*Details for the centers*		
	
	*UCTH*	*FMCO*	*NAUTH*
Generator type	GEC MX-4	Digital Visitor AR30	Easymatic Super 325
	1-Ø (medium freq)	3-Ø, 300 mHz	(Universal UX) 1-Ø, 60 Hz
Age	>20 years	3 years	>10 years
Focal spot size	0.5/1.0	0.6/1.2	1.0/2.0
Filtration (total)	2.6mm Al eq.	2.5mm Al eq.	2.7 mm Al eq.
Tube output/reproducibility (mGy)	1.84 (σ = 0.11)	0.16 (σ = 0.01)	1.65 (σ = 0.17)
Film speed	200	200	200

1-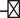 = single phase; 3-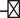 = three phase. Tube output was determined by determining ratio of absorbed dose to TLD over five exposures, UCTH - University of Calabar Teaching Hospital, NAUTH - Nnamdi Azikiwe University Teaching Hospital

**Table 2:** Patient criteria and average radiographic factors recorded in the study

*Projection*	*Criterion*	*Hospital/centers*		
		
		*UCTH*	*FMCO*	*NAUTH*
Chest (AP/PA)	No. of patients	37	26	36
	No. of males	16	14	21
	No. of females	21	12	15
	Age (years)	2.13 (0-9)	2.57 (0-9)	2.25 (0-6)
	kV	70.04 (60-84)	50.8 (50-55)	49.89 (48-50)
	mAs	28.91 (10-80)	1.39 (0.3-6.4)	25.66 (15-45)
	FFD	104.17 (100-150)	105.6 (100-150)	107.9 (100-150)
Abdomen	No. of patients	5	5	5
	No. of males	2	4	3
	No. of females	3	1	2
	Age (years)	3.56 (0-10)	2.84 (0-10)	0.45 (0-1)
	kV	71.67 (70-75)	52.5 (50-65)	50
	mAs	40.8 (30-62.4)	3.72 (2-8)	50 (30-75)
	FFD	96.7 (90-100)	92.5 (90-100)	96.7 (90-100)
Skull (AP/Lat)	No. of patients	10	8	16
	No. of males	3	6	12
	No. of females	7	2	4
	Age (years)	3.43 (1-9)	1.41 (0-2)	1.56 (0-2)
	kV	78.4 (75-88)	50	50
	mAs	58.6 (50-62.5)	2.13 (2-3.2)	43.5 (30-45)
	FFD	95 (90-100)	94.4 (90-100)	91 (90-100)
L. Spine	No. of patients	n.a	6	11
	No. of males	-	3	8
	No. of females	-	3	3
	Age (years)	-	0.75 (0-2)	0.15 (0-1)
	kV	-	50	50
	mAs	-	4 (2-10)	22.5
	FFD	-	95 (95-100)	92.5 (90-100)
Pelvis	No. of patients	8	11	11
	No. of males	3	4	3
	No. of females	5	7	8
	Age (years)	0.53 (0-1)	0.5 (0-1)	0.56 (0-1)
	kV	67.3 (60-84)	50	47.5 (40-50)
	mAs	23.5 (12-45)	4.14 (2-8)	23.4 (15-45.5)
	FFD	95 (90-100)	94.3 (90-100)	91.3 (90-100)

The range of the variables is indicated by the figures in parenthesis, UCTH - University of Calabar Teaching Hospital, NAUTH - Nnamdi Azikiwe University Teaching Hospital

LiF TLD disks (0.3 mm thick) were calibrated for sensitivity and linearity at the Secondary Standard Dosimetry Laboratory of the Federal Radiation Protection Services (FRPS), Ibadan. Calibration was done with known doses of X-rays at kVp 50-100, in 5 kVp steps, to obtain a calibration factor used to convert thermoluminesecence (TL) intensity to dose. Irradiated TLDs were read with a Vinten Solaro Model 680 reader at the same place. The mean deviation between the detectors was ±5%. The TL detectors were placed in radioparent cellophane bags with negligible radiation attenuation property to prevent accumulation of dirt and grease from handling. Dose output and reproducibility of the X-ray units, tested with TLDs over five exposures at 65 kVp and 12 mAs, preceded ESD collation. ESDs were obtained by placing the TLD discs at the center of the X-ray beam incident on the patients' skin. ESDs determined in this study were compared with values reported in literature for three hospitals in another region of Nigeria, as well as in published data for some hospitals in Sudan.

## Results

Sample sizes in some examinations were very small (<10), as shown in Table 3. However, doses for such examinations are reported because of the difficulty in obtaining sufficient number of patients for each category. Despite the high level of error introduced by these small samples, the doses are reported because this work is the first attempt at estimating effective doses in the area of study, and it is therefore believed that the data is essential for comparison with future measurements.

**Table 3:** Distribution of patients by age range for each examination across the hospitals

*Projection*	*Age range*	*UCTH*	*FMCO*	*NAUTH*
Chest	0-1	16	10	14
	1-5	12	9	10
	5-10	9	7	12
Abdomen	0-1	3	2	5
	5-10	-	3	-
	10-15	2	-	-
Skull	0-1	-	4	9
	1-5	6	-	7
	5-10	4	4	-
L. spine	0-1	-	3	11
	1-5	-	3	-
Pelvis	0-1	8	11	11

UCTH - University of Calabar Teaching Hospital, NAUTH - Nnamdi Azikiwe University Teaching Hospital

The range of doses recorded for tube output and reproducibility were 0.15-0.17 (FMC), 1.48-1.89 (NAUTH) and 1.69-1.98 (UCTH) for the same exposure factors and using the same TLD discs over the five exposures.

ESD values obtained are shown in Table 4. As reported elsewhere, there were significant differences in the values obtained for the different hospitals, for the same examinations and same age range of the patients. Differences also exist for data for the same examination within the same hospital. The results are reported for different age ranges and sex [Table 4] for X-ray projection monitored in each hospital.

**Table 4:** Mean entrance skin dose ± σ (mGy) values for each hospital by examination and age range

*Center*	*Age group*	*Radiographic projection/ESD ± σ (mGy)*				
		
		*Chest (PA/AP)*	*Abdomen*	*Skull (AP/Lat)*	*Spine (Lumbar)*	*Pelvis*
UCTH	0-1	0.64 ± 0.15	1.83 ± 0.12	-	-	1.82 ± 0.81
	1-5	1.82 ± 1.17	-	6.22 ± 0.95	-	-
	5-10	1.70 ± 1.04	-	5.15 ± 1.77	-	-
	10-15	-	6.60 ± 0	-	-	-
FMCO	0-1	0.07 ± 0.01	0.13 ± 0.03	0.11	0.14 ± 0.02	0.07 ± 0.08
	1-5	0.12 ± 0.1	-	0.10 ± 0.04	0.4 ± 0	-
	5-10	0.14 ± 0.1	0.40 ± 0	-	-	-
	10-15	-	-	-	-	-
NAUTH	0-1	1.10 ± 0.1	1.20 ± 0.13	1.34 ± 0.2	1.18 ± 0.4	0.74 ± 0.3
	1-5	1.04 ± 0.3	-	1.32 ± 0.2	-	-
	5-10	1.10 ± 0.1	-	-	-	-
	10-15	-	-	-	-	-

s: standard deviation, UCTH - University of Calabar Teaching Hospital, NAUTH - Nnamdi Azikiwe University Teaching Hospital

ESDs were the highest in UCTH for patients aged 1-10 years presenting for chest and skull radiography respectively. Doses recorded for the age range 0-1 year were higher for UCTH than doses in the other two hospitals for pelvis. Abdominal doses for ages 1-5 and 5-10 in UCTH were very much higher than the values reported in literature.[4,20] The applied technique needs to be examined.

Distribution of the ESDs according to gender shows that in most cases, as presented in Table 5, there were differences in the values obtained between male and female patients within the same hospital and for the same examinations. Generally, ESDs for the female patients were higher than their male counterparts. No reason was given by the radiographers for this disparity. This is currently being further investigated. However, differences in technique by individual operators, as well as differences in patient body mass index (due to age range divisions used), may contribute to this.

**Table 5:** Distribution of mean entrance skin dose ± σ (mGy) by sex

*Projection*	*Age range*	Sex	*Mean ESD ± σ (mGy) per hospital*		
			
			*UCTH*	*FMCO*	*NAUTH*
CXR (PA/AP)	0-1	M	0.50	0.06	1.09 ± 0.16
		F	0.70 ± 0.17	0.07 ± 0.01	1.08 ± 0.13
	1-5	M	1.87 ± 1.42	0.11 ± 0.1	1.02 ± 0.28
		F	2.10 ± 1.42	0.17 ± 0.12	1.05 ± 0.19
	5-10	M	0.80	0.27 ± 0.16	1.30
		F	2.60	0.04 ± 0.01	1.05 ± 0.10
	10-15	M	-	-	1.00
		F	-	-	
Abdomen (AP)	0-1	M	1.90	0.14 ± 0.03	1.26 ± 0.20
		F	1.70	0.1	1.2
	1-5	M	-	-	-
		F	-	-	-
	5-10	M	-	0.40	-
		F	-	-	-
	10-15	M	-	-	-
		F	6.60	-	-
Skull	0-1	M	-	0.11	1.36 ± 0.18
		F	-	-	-
	1-5	M	5.55 ± 1.77	0.09 ± 0.02	1.37 ± 0.15
		F	6.56 ± 0.12	0.13 ± 0.07	1.32 ± 0.18
	5-10	M	3.00	-	-
		F	5.50 ± 1.56	-	-
	10-15	M	-	-	-
		F	-	-	-
L. spine	0-1	M	-	0.13 ± 0.02	1.22 ± 0.40
	-	F	-	0.15	0.95 ± 0.07
	1-5	M	-	-	-
	-	F	-	-	-
	5-10	M	-	-	-
	-	F	-	-	-
	10-15	M	-	-	-
Pelvis	0-1	M	1.35 ± 0.92	0.24 ± 0.07	0.47 ± 0.19
		F	1.75 ± 1.08	0.12 ± 0.04	0.87 ± 0.30
	1-5	M	-	-	-
		F	-	-	-
	5-10	M	-	-	-
		F	-	-	-
	10-15	M	-	-	-

UCTH - University of Calabar Teaching Hospital, NAUTH - Nnamdi Azikiwe University Teaching Hospital, ESD - Entrance skin dose

## Discussion

The purpose of any radiological procedure is ultimately to achieve the well-being of the patient. Children undergoing X-ray examination in three hospitals in eastern Nigeria were monitored to obtain the entrance surface doses for the commonest and most frequent examinations in the hospitals. The higher life expectancy of the child patient makes it necessary to ensure optimization in the procedures used. This survey has revealed high ESDs in the area of study.

**Figure 1:**
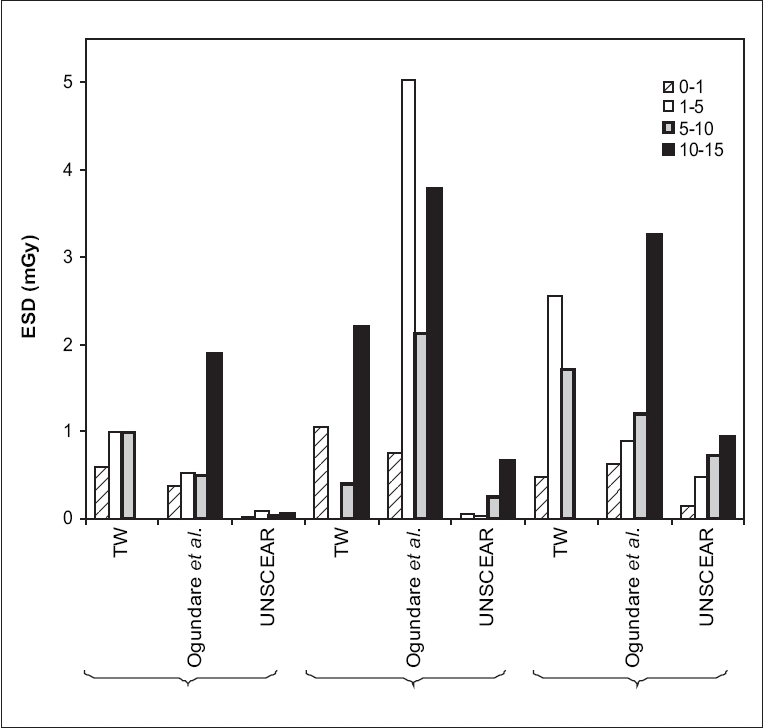
Comparison of mean ESDs for the three hospitals, for chest, abdomen and skull in this work (TW) with data from three other hospitals in Nigeria from the study by Ogundare *et al*.,[19] and from UNSCEAR.[20] The age range 0-1 month was merged with 1 month - 1 year for this comparison. The values plotted in the figure are therefore mean values for the two age groups (0-1 month and 1 month - 1 year) in the references[19] and,[20] respectively.

Figure 1 is a comparison of the results of this study with those of the study by Ogundare *et al.*;[19] and with data from United Nations Scientific Committee on the Effects of Atomic Radiation (UNSCEAR)[20] for chest, abdomen and skull radiographic projections in children. Generally, the high values of ESDs obtained are in agreement with the results from an earlier work covering three hospitals in the western part of Nigeria.[19]

ESDs for chest radiography in children, recorded in this work are higher than the values obtained in reference[19] by about 36.6% for ages 0-1 year, 47.5% for ages 1-5 years and 50% for ages 5-10 years respectively. The mean percentage difference in ESD for chest radiography for these two studies is about 44.7%. For the abdominal radiographs, the results from this study are higher than those reported earlier by 28.6% for age group 0-1 year. ESDs for skull films were 65.1% (for 1-5 years) and 29.8% (for 5-10 years) higher than those reported in the cited work.[19] However, lower ESDs were recorded for the abdominal examinations of age groups 5-10 years and 10-15 years, as well as for skull examinations of children aged between 0 and 1 year in the current study. These results show pediatric doses in the area of study to be higher than the values obtained in western Nigeria. The ESDs are also above values obtained in the three hospitals in Sudan,[21] as well as the reference levels recommended by the National Radiological Protection Board (NRPB)[4] and UNSCEAR.[20] One reason for this difference may be due to the influence of the Federal Radiation Protection Service, cited in the west, on hospitals within that catchment area. Widespread coverage of the activities of this institution is hampered by several factors, including lack of sufficient manpower, adequate equipment and logistics arising from other economic and political problems. It is hoped that the recent establishment of the Nigerian Institute for Radiation Protection and Research (NIRPR) will raise the profile of radiation protection and facilitate the necessary evolution of dosimetric reference parameters for the country.

The obtaining of high ESDs is attributed to the use of low kilovolts; and in the case of UCTH and NAUTH, very high milliampere-second (mAs) values [Table 2]. The state of some of the equipment used in the departments is a source of concern. Apart from FMC, which has relatively new equipment, the other facilities studied have an average age greater than 15 years. Emission of soft radiation during the pre-contact phase in old generators is common and can add to the ESD, especially in the absence of added filtration. Additional tube filtration is not used in any of the hospitals, despite the fact that all the X-ray units have relatively low inherent tube filtration. The wide range of doses obtained for tube reproducibility may be an indication of a lack of accuracy of either kVp meter or timer in UCTH and NAUTH units. Testing of these units with appropriate equipment has been recommended to confirm accuracy as this may be a major contributor to the high doses recorded in these centers.

The wide variation in doses obtained for patients within the same hospital for similar examinations is the result of lack of any form of standardization in the procedures. There are therefore as many techniques as there are different radiographers in any one system. There is also no evidence of quality control checks on the X-ray generators to determine their level of reproducibility. Urgent attention needs to be paid to these concerns. Film processing conditions could also be a reason for the high doses. The lack of sensitometric studies for the manual processing techniques in the three hospitals often tells on the quality of radiographs produced, with radiographers sometimes having to compensate for weak processing solutions by increasing exposure factors.

While these results may not be the position of all diagnostic centers across the country, the findings suggest that very urgent steps need to be taken to address dose reduction in the three hospitals. Optimization of dose or resetting the exposure parameters so that minimum possible dose may be delivered to patients without compromising the image quality is urgently recommended in the centers recording the highest doses.

For this, quality assurance (QA) programs must be set up and executed on regular basis. This has been shown to have significant effect in reducing doses and improving image quality.[22] It is hoped that the NIRPR will facilitate increased intra-/inter-hospital surveys and monitoring of doses and subsequently develop radiation protection reference dose levels for the country. This work will serve as a starting point for dose monitoring in the region surveyed.

## Conclusion

ESD measurements on children undergoing radiological examinations in three Nigerian hospitals show doses very much higher than recommended values. There is therefore urgent need to cut these doses. To this end, the generally practiced high kilovoltage technique and lower mAs are recommended, along with a change in the speed of the intensifying screens and films used. The development of a QA program to address the lapses in equipment and technical performance, either centrally or in each hospital, will go a long way in reducing ESDs in these hospitals, which will be evident from future monitoring.
